# Strategies to achieve adequate vitamin A intake for young children: options for Cameroon

**DOI:** 10.1111/nyas.14275

**Published:** 2019-12-03

**Authors:** Stephen A. Vosti, Justin Kagin, Reina Engle‐Stone, Hanqi Luo, Ann Tarini, Adrienne Clermont, Jules Guintang Assiene, Martin Nankap, Kenneth H. Brown

**Affiliations:** ^1^ Department of Agricultural and Resource Economics University of California, Davis Davis California; ^2^ Kagin's Consulting Vacaville California; ^3^ Department of Nutrition University of California, Davis Davis California; ^4^ Ann Tarini International Public Health Consulting Montreal Quebec Canada; ^5^ Johns Hopkins Bloomberg School of Public Health Baltimore Maryland; ^6^ Helen Keller International Yaoundé Cameroon; ^7^ UNICEF Yaoundé Cameroon

**Keywords:** vitamin A, children, dietary intake, modeling, cost‐effectiveness, policy pathways, Cameroon

## Abstract

Meeting children's vitamin A (VA) needs remains a policy priority. Doing so efficiently is a fiscal imperative and protecting at‐risk children during policy transitions is a moral imperative. Using the Micronutrient Intervention Modeling tool and data for Cameroon, we predict the impacts and costs of alternative VA intervention programs, identify the least‐cost strategy for meeting targets nationally, and compare it to a business‐as‐usual (BAU) strategy over 10 years. BAU programs effectively cover ∼12.8 million (m) child‐years (CY) and cost ∼$30.1 m; ∼US$2.34 per CY effectively covered. Improving the VA‐fortified oil program, implementing a VA‐fortified bouillon cube program, and periodic VA supplements (VAS) in the North macroregion for 3 years effectively cover ∼13.1 m CY at a cost of ∼US$9.5 m, or ∼US$0.71 per CY effectively covered. The tool then identifies a sequence of subnational policy choices leading from the BAU toward the more efficient strategy, while addressing VA‐attributable mortality concerns. By year 4, fortification programs are predicted to eliminate inadequate VA intake in the South and Cities macroregions, but not the North, where VAS should continue until additional delivery platforms are implemented. This modeling approach offers a concrete example of the strategic use of data to follow the Global Alliance for VA framework and do so efficiently.

## Graphical abstract

The objectives of this paper are to apply the Micronutrient Intervention Modeling tool to: update the model with a revised set of vitamin A (VA) intervention programs and data to support them, and identify concrete subnational policy pathways (sequences of VA program modifications) in Cameroon, which are guided by the Global Alliance for Vitamin A framework.

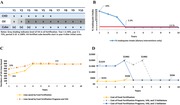

## Introduction

Vitamin A deficiency (VAD) affects hundreds of thousands of children globally, particularly in low‐ and middle‐income countries (LMIC),^1^ and increases their risk of morbidity and mortality.^2^ Results from large randomized, controlled trials have indicated that vitamin A supplementation (VAS) of children conducted in areas with a high risk of VAD reduces mortality.^3^, ^4^, ^5^ Based on these results, national programs have been implemented in multiple countries to provide children with high‐dose VAS distribution semiannually. In many countries, VAS distribution has been linked to vaccination campaign platforms, such as Child Health Days (CHD).^6^ These programs were initially intended as a short‐term solution, to mitigate the loss of life, while other medium‐ and long‐term strategies to address underlying VAD and low intake were put in place, but have now been operating for decades in many countries. While some countries have made progress in addressing VAD through other programs, such as fortification (e.g., Guatemala^7^), many countries either do not have such alternative programs in place, or the programs are not functioning well.^8^, ^9^ At the same time, as vaccine‐preventable diseases such as polio near eradication, funding for the delivery platforms that have been used for VAS distribution has been reduced.^10^ Some countries (e.g., Ethiopia) have switched to other delivery platforms for VAS, such as during routine healthcare contacts, but these systems are often weak in low‐income settings. Countries that lose funding for twice‐annual VAS distribution but do not yet have other systems in place for VAS distribution or other vitamin A (VA) programs will be at risk of experiencing increased child mortality.

In countries where VA programs, such as food fortification, have been implemented, the Global Alliance for Vitamin A (GAVA) proposed a framework for deciding when to scale back VAS distribution;^11^, ^12^ this framework was recently updated.^13^ The original framework relies on collecting appropriate evidence to indicate that programs to increase daily VA intake are functioning well and that VAD has been reduced below selected thresholds. Specifically, the framework recommends that countries (1) collect dietary data and program monitoring data to assess whether programs are working well and diets are improving; (2) conduct a survey using VA biomarkers among children (hereafter, referred to as a validation study) to assess whether the prevalence of VAD (typically measured using serum retinol) has decreased; (3) if the prevalence of VAD is <5%, conduct a second validation study in a nonconsecutive year to confirm the change in VA status; and (4) if the decrease in VAD is confirmed, withdraw the VAS program, with appropriate monitoring during the transition.^12^ The new framework relaxes these conditions somewhat by recommending that VAS program scale back be considered in populations where the prevalence of VAD (determined using biomarkers) is <10% and dietary intake is shown to be adequate for the population.^13^ Both versions of the framework are intended for use once alternative VA programs are up and running, but they do not include guidance for countries on how to select or implement the appropriate program, or combination of programs, to reach the point where VAD is likely to have decreased and the GAVA framework can be applied to policy discussions about withdrawing VAS.

The Micronutrient Intervention Modeling project (MINIMOD) aims to fill this gap by applying a tool composed of three models (nutrition benefits, program costs, and economic optimization) to design and manage micronutrient (MN) intervention programs more efficiently. By predicting the costs and likely health and nutrition impacts of selected existing and new programs, the tool provides information that can be introduced into policy discussions to improve the cost‐effectiveness of national and subnational MN strategies. Previously, the MINIMOD tool was applied to the case of VA programs in Cameroon (where baseline VAD rates among young children ranged from over 40% inflammation‐adjusted retinol‐binding protein <0.83 µmol/L in the North macroregion to less than 25% in the South macroregion), to identify the most cost‐effective mix of programs that would achieve the same effective coverage as current (business‐as‐usual, BAU) programs (VAS, deworming (DW), and fortified edible oil).^14^, ^15^, ^16^, ^17^ VA‐related programs introduced into the MINIMOD tool included high‐dose VAS distributed via CHD, multiple MN powders also distributed via CHD, large‐scale food fortification, and VA‐biofortification of maize.^1^ The results suggested that modifying the composition of programs and shifting the geographic focus of VA programs to the North macroregion (where the burden of VAD was greatest and where VAS programs were most efficient) could achieve the same effective coverage as the current portfolio, at approximately 50% lower cost.^17^

The objectives of this paper are to apply the MINIMOD tool to update the model with a revised set of VA intervention programs and data to support them, and to identify concrete subnational policy pathways (sequences of VA program modifications) in Cameroon, which are guided by the GAVA framework.^2^ More specifically, we (1) apply the MINIMOD tool to estimate the impacts, costs, and cost‐effectiveness of policy‐relevant VA intervention programs over 10 years for three ecological macroregions of Cameroon; (2) apply the MINIMOD tool's economic optimization model to identify *the* most cost‐effective strategy for meeting VA nutrition objectives *nationally*; and then (3) identify macroregional, year‐by‐year policy pathways (programmatic changes) leading from a BAU scenario toward more efficient sets of policies that achieve adequate VA intake while protecting children from VAD‐related mortality during policy transitions.

## Methods

Previously, we used the MINIMOD tool's nutrition benefits model to predict the effects of alternative VA intervention programs, and combinations thereof, on dietary VA adequacy^15^ and lives saved.^18^ Using the tool's cost model,^16^ we estimated program costs over 10 years, including start‐up costs of all VA intervention programs and combinations of them. We then applied the MINIMOD tool's economic optimization model^17^ to identify the most cost‐effective strategy to meet VA needs over 10 years, compared with programs in place under a BAU strategy. For our analysis here, we updated the results of the foregoing analyses with a slightly different set of VA interventions and used the economic optimization model to identify practical *macroregion–specific* policy pathways that lead from the BAU strategy to a more efficient set of VA programs, but constrained to adhere to the original GAVA framework. Finally, we calculated the costs associated with adhering to the GAVA protocol.

### VA intervention programs modeled

A broad array of programs and policies exist that have the potential to directly (e.g., via food fortification) or indirectly (e.g., price policies that incentivize the consumption of VA‐rich foods) increase VA intake. Decision makers in Cameroon were most interested in a subset of programs that were underway or were being discussed as possible additional/replacement interventions. More specifically, a fortification program for edible oils (consumed on the previous day by 39% (South macroregion) to 79% (Yaoundé/Douala)^19^ of children nationally, and fortified with VA) was ongoing but could be strengthened, a VA‐biofortified maize program was being considered (any maize consumption by children on the previous day ranged from 34% (Yaoundé/Douala) to 45% of children (North macroregion)^19^), as was the fortification of bouillon cubes (consumed on the previous day by >86% of children in each macroregion).^3^ In addition, twice‐annual distribution of high‐dose VAS has been ongoing in Cameroon for decades, with reach varying regionally and over time. DW treatment is also included in the programs in Cameroon, but we have excluded this from the current calculations due to the low hypothetical contribution to VA status.

### Estimation of VA intervention program benefits

The target population for VA intervention programs in Cameroon is children 6–59 months of age. We calculated three indicators of program impact (reach, effective coverage,^15^ and lives saved^18^). Reach was defined as the proportion of individuals who consumed *any* additional VA because of a given program, regardless of how much additional VA was consumed, or whether an individual had low VA intake or status. Effective coverage was defined as the proportion of individuals who had inadequate dietary VA intake and subsequently achieved adequate intake as a result of one or more VA intervention programs. Effective coverage was calculated by estimating the prevalence of dietary VA adequacy at baseline (intake from typical diet alone) and under different programmatic scenarios (e.g., edible oil or bouillon cube fortification, high‐dose VAS administration) to calculate the *increase* in proportion of individuals with adequate VA intake. The Lives Saved Tool (LiST) model^20^ was used to estimate the effects on VA‐related child mortality of alternative VA intervention programs, and combinations of them.^18^

The data used to estimate the baseline prevalence of inadequate nutrient intakes were collected during a 2009 national survey of MN status and dietary intake in Cameroon, stratified at the level of three macroregions: North, South, and Cities (Yaoundé and Douala).^19^, ^21^ The dataset includes at least 1 day of dietary intake data for all respondents, and a second day of data for a subset of respondents, to enable estimation of the distribution of habitual (as compared with a single‐day) dietary intake.^22^ We applied the National Cancer Institute (NCI) method^23^, ^24^ to generate the distribution of usual nutrient intakes and estimate the prevalence of inadequate intake (i.e., proportion with intakes below the age‐ and sex‐specific estimated average requirement ) at baseline (i.e., in the absence of any MN intervention programs, such as fortification or supplementation). Nationally, roughly half of children in this age group suffered from inadequate VA intake; however, this national average masks substantial macroregional variation, ranging from 35% in the South macroregion to 73% in the North macroregion. Notably, these regional patterns of the prevalence of inadequate VA intake were similar to the observed patterns of VA deficiency (defined as low inflammation‐adjusted plasma retinol‐binding protein concentrations or as low breast milk VA concentrations),^14^, ^25^ in that both dietary intake and biomarkers identified the population in the North macroregion as having the greatest risk of low VA intake and status.

We simulated the effects of VA intervention programs, and combinations of them, by calculating the additional daily VA intake contributed by one or more programs and adding this to each person's baseline intake. The specific method applied for each type of intervention program is described below. We then reapplied the NCI method to estimate the postintervention distribution of usual intakes and prevalence of inadequate intake under each scenario.^26^ Finally, effective coverage was calculated as the difference between prevalence of inadequate intake in each modeled scenario compared with the baseline.

To estimate the contribution of fortified edible oil to dietary VA intake, we multiplied fortifiable oil consumption (grams per day) by the assumed fortification level. Two scenarios were modeled: (1) implementation of VA fortification at 44% of the target level of 12 mg/kg of oil, based on measurements of fortified oil samples from a program evaluation;^21^ and (2) an enhanced program, where, through additional monitoring and enforcement activities, the oil fortification levels are increased gradually from 44% of target to 100% of the target level over a period of 3 years. Fortified bouillon cube impacts were estimated in the same way, under the assumption that 3 years of program planning would be necessary before 100% of bouillon cube would be fortified (80 mg/kg of bouillon cube) beginning in year 4 of the simulation.

To estimate the contribution of biofortified maize, we assumed that the consumed product contains 0.34 µg of beta‐carotene RAE per kcal,^27^ and that after a 3‐year phase‐in period during which nationwide investments are made in agroecozone‐specific varietal improvements and agricultural extension, all maize in the national marketplace is biofortified at that level.^4^

To estimate the contribution of high‐dose VA supplements to dietary VA adequacy, a daily intake equivalent of 167 µg retinol activity equivalents per day (RAE/day) was developed;^15^ this amount was added to the baseline VA intake for all children who reportedly received a VA supplement during the 6 months prior to the dietary survey. The reach of VAS was assumed to remain the same as that measured in the dietary survey (64% South, 89% North, and 58% Yaoundé/Douala).

#### Linkage with LiST

We estimated the number of child deaths averted through VA intervention programs by introducing effective coverage estimates into Lives Saved Tool (LiST)^20^ through an adapted method that has been described previously.^18^ Briefly, LiST projection files were created for each of the three macroregions to estimate the cause of death structure among children <5 years of age. The LiST tool, as originally developed, estimates the contribution of high‐dose VAS (but not other VA interventions) to reductions in child mortality. We modified the parameters in the original LiST method to predict the effect of achieving adequate VA intake (effective coverage) on VA‐related child mortality. Specifically, we substituted effective coverage estimates for VAS coverage (i.e., receipt of a capsule) and derived an estimate of the effectiveness of achieving dietary adequacy for reducing diarrheal deaths, the main contributor to VA‐preventable child deaths in LiST. To estimate the total number of VA‐preventable deaths in each macroregion, the baseline coverage of VAS in LiST was first rolled back to zero to simulate a scenario in which no VA interventions are in place. From this baseline coverage of zero, VA programs are then scaled up in the LiST projection file by using the effective coverage estimates for various packages of VA interventions (generated through the dietary modeling described above) to calculate deaths averted in each scenario.

### Estimation of VA intervention program costs

To estimate the costs^5^ of VA programs in Cameroon, we used recent budgets from current programs, when available.^16^ When program budgets were not available, for example, for experimental delivery platforms or planned improvements to existing platforms, we estimated costs based on known unit costs of program components and expert knowledge distilled from a cost workshop convened in Cameroon in April, 2014.^16^

#### Large‐scale food fortification

Costs of large‐scale fortification programs were drawn primarily from budgets created by Helen Keller International‐Cameroon. Program costs included program planning costs, up‐front investments required to outfit edible oil filtering/containerizing production processes to blend in fortificants, the costs of fortificants, and the private and public costs associated with program monitoring and evaluation. The costs associated with designing and implementing a fortified bouillon cube program, including a 3‐year start‐up period during which costs would be incurred but no nutritional benefits would be generated, were based on input provided at a 2014 cost workshop by members of the food industry and organizations that designed and managed the national edible oil and wheat flour fortification programs.

In some of the results that follow, we examined the potential effects of improving the efficiency and effectiveness of current large‐scale fortification programs, by increasing investments in the monitoring and evaluation of programs to ensure full industry compliance.^6^

#### Biofortification of maize

Biofortification refers to the process of selectively breeding varieties of staple crops so that they contain higher‐than‐average, bioavailable amounts of key MNs.^27^ The data for maize biofortification program cost estimates were drawn from budgets developed during a workshop involving experts from Cameroon on biofortification. The very large up‐front research and development costs associated with fundamental trait selection and plant breeding are generally paid by the international community (e.g., by one of more of the centers that comprise the Consultative Group on International Agricultural Research–CGIAR), and hence were not included as costs to national biofortification programs. However, program costs included in‐country varietal selection and agronomic improvement activities, as well as the costs associated with seed multiplication and agricultural extension programs required to ensure the availability and proper use of new planting material. No nutritional benefits were assumed to emerge from biofortification programs during the first 3 years of their operation.

#### VAS distribution through child health days

CHDs in Cameroon target all children 6–59 months of age, regardless of their health or nutritional status.^7^ These twice‐annual CHD campaigns are large and expensive.^16^ The data used to estimate the costs of CHD were primarily drawn from budgets created by United Nations Children's Fund‐Cameroon and Helen Keller International‐Cameroon for the districts they managed during the second round of the CHD campaign in 2012.^8^ Cost categories included, among other things: (1) the distribution of the VA capsules to the children through door‐to‐door visits; (2) VA capsule costs, including freight and delivery costs; (3) campaign personnel supervision, per diem, transportation, and other costs; (4) training costs; (5) TV and radio communication costs; (6) costs of collecting and processing program evaluation data; and (7) national‐level administrative costs.

An array of products and services are generally provided during CHDs, for example, the distribution of high‐dose VAS and DW tablets, catch‐up immunizations, and sometimes oral polio vaccine. For the purposes of this paper, we focused only on VAS. To isolate the costs of these interventions, we first removed costs directly related to vaccinations, for example, vaccination teams, vaccination training, vaccine costs, and so on. We then assumed that 90% of the overhead costs associated with CHDs would have to be covered by the VAS program.^9^^,^ 10

#### Economic optimization model to identify the most cost‐effective set of VA intervention programs

We used the MINIMOD economic optimization model to identify the most cost‐effective strategy for addressing inadequate VA intake among young children.^17^ More specifically, the model uses a mixed integer linear programming approach that aims, in the results presented below, to effectively cover at least the same number of child‐years (CY)^11^ (nationally) over 10 years as the BAU scenario,^12^ but including a broader array of candidate VA intervention programs. The set of candidate programs included in the optimization algorithm were, among others: current VA‐fortified edible oil program, enhanced VA‐fortified edible oil programs, VA‐fortified bouillon cubes, VA‐biofortified maize, and VAS programs that can be deployed at the macroregional level. The model was free to shift resources across VA intervention programs, across macroregions, and over time with the objective of seeking the most cost‐effective collection of programs that can meet (at least) the *national* BAU programs’ outcomes, namely CY effectively covered and lives saved over the *entire* 10‐year planning time horizon. The BAU programs were composed of a national VA‐fortified edible oil program that nationally delivers approximately 44% of the targeted amount of VA and a national VAS program delivered via CHD.^13^

#### Policy pathways for improving the efficiency of VA programs

The MINIMOD economic optimization model will identify the *most* cost‐effective combinations of VA intervention programs nationally over 10 years for meeting a prespecified target, in this case, the number of CY effectively covered by the BAU programs. However, transitioning from the BAU set of programs to the economically optimal set of programs will not be immediate or free of additional costs; a variety of technical, political, and other issues will also need to be addressed, and investments may be required to upgrade existing programs and to put new programs in place. Depending on the issues to be addressed and the program upgrades to be made, progress can be quicker or slower, and progress can vary by macroregion. Moreover, achieving the *nationally* economically optimal solution will generally involve trade‐offs across macroregions regarding where cases of VAD are resolved; by design, the model takes advantage of macroregional differences in VAD and region‐specific differences in the cost‐effectiveness of alternative VA intervention programs in addressing VAD. It is possible that some trade‐offs suggested by the model may be deemed unacceptable for political or other reasons, and objectives may be established for macroregions rather than for the entire nation.

Therefore, it is important to identify *policy pathways* (sequences of policy changes that lead from the BAU set of VA intervention programs towards a final set of more efficient programs) that address these issues, and thereby generate political support for medium‐term strategies for reducing MN deficiencies, and help secure financial support for these transitions. The MINIMOD tool allows for the examination of multiple pathways leading from the BAU strategy toward the economically optimal set of VA intervention programs for each macroregion, on an annual time‐step. In what follows, we use the MINIMOD tool to examine one such pathway for *each* of Cameroon's macroregions separately,^14^ with the added condition that the pathway adhere to the GAVA protocol for assessing the wisdom of scaling back VAS programs. More specifically, the tool seeks the most cost‐effective collection of VA intervention programs but requires the retention of VAS programs until VAD among children is confirmed to fall below an agreed‐upon level. For this analysis, the dietary threshold we adopted for triggering a validation study to confirm decrease in prevalence of VAD was the prevalence of inadequate intake below 2.5% among children 6–59 months of age, representing the theoretical target for food fortification programs.^28^ Moreover, the tool is required to retain the VAS program for up to 3 additional years, during which validation studies are undertaken to confirm that the prevalence of biochemically defined VAD has been reduced to levels at which VAD is no longer considered a public health problem. We also calculate the difference in costs of a departure from the original GAVA recommendations; namely, if it is only feasible (politically, financially, or otherwise) to conduct a single validation study compared with two validation studies.

#### Model robustness tests

There are two (summary) sources of uncertainty in the MINIMOD tool: the estimated nutritional benefits and the estimated costs of alternative VA intervention programs. To assess the extent to which these sources of uncertainty affect the programs chosen by the economic optimization model, probability distributions around the point estimates of benefits and costs were constructed, and Monte Carlo simulations involving 1000 simulations based on random draws from those distributions were performed. Probability distributions were constructed using standard errors for effective coverage estimates (assuming a normal distribution); for costs, uniform distributions of costs ±20% were constructed to generate 95% intervals.

## Results

For each of the VA intervention programs considered, the projected 10‐year^15^ reach, effective coverage, lives saved, and cost‐effectiveness measures for each of these outcomes varied by intervention and macroregion (Table 1).

**Table 1:** National and subnational predicted nutritional impacts, costs, and cost‐effectiveness of selected vitamin A programs over 10 years

	Reach, '000s of child‐years^a^	Effective coverage, '000s of child‐years	Child deaths averted, number of children	Total cost,^f^ '000s US$	Cost per child reached, US$	Cost per child‐year effectively covered, US$	Cost per child death averted, US$
VA‐fortified edible oils (44% target)^b^							
National	17,188	5075	9724	$2657	$0.15	$0.52	$273
North^c^	7135	1459	3836	$928	$0.13	$0.64	$242
South	5435	1960	3577	$1015	$0.19	$0.52	$284
Cities	4618	1657	2311	$713	$0.15	$0.43	$309
VA‐fortified edible oils (44–100% target)^c^							
National	17,188	8055	15,527	$4851	$0.28	$0.60	$312
North	7135	2669	8354	$1695	$0.24	$0.64	$203
South	5435	3141	4862	$1853	$0.34	$0.59	$381
Cities	4618	2246	2311	$1303	$0.28	$0.58	$564
VA‐fortified bouillon cubes^d^							
National	29,039	7731	16,098	$2932	$0.10	$0.38	$182
North	10,958	3300	8653	$1094	$0.10	$0.33	$126
South	12,820	2940	5365	$1195	$0.09	$0.41	$223
Cities	5261	1491	2080	$643	$0.12	$0.43	$309
VA‐biofortified maize^e^							
National	13,435	2512	5720	$1398	$0.10	$0.56	$244
North	5734	1512	3975	$618	$0.11	$0.41	$155
South	5713	808	1476	$593	$0.10	$0.73	$402
Cities	1988	192	269	$187	$0.09	$0.97	$696
VA supplementation via child health days							
National	23,649	8586	19,267	$26,923	$1.14	$3.14	$1397
North	11,340	5201	13,630	$8766	$0.77	$1.69	$643
South	8918	2131	3889	$12,963	$1.45	$6.08	$3333
Cities	3391	1253	1748	$5194	$1.53	$4.15	$2972

a The total number of child‐years over the 10‐year model timeline is national 32.5 m, North 12.7 m, South 13.9 m, and Cities 5.8 m.

b Indicates measured oil fortification levels in 2012.^20^ VA, vitamin A.

c North: Extreme North, North and Adamawa regions; South: South, East, Centre, Littoral, West, Southwest, and Northwest regions (excluding Yaoundé and Douala); Cities: Yaoundé and Douala.

d Indicates that the fortified oil program is strengthened such that the VA content of oil increases over a 3‐year period from 44% to 100% of target fortification levels.

e Ten‐year averages are reported, even though benefits do not begin to accrue until year 4 of the simulation period (program is assumed to be implemented at 100% of target beginning in year 4).

f All costs are reported in 2013 USD; exchange rate applied was 500 CFA = 1 USD.

In terms of program reach, the VA‐fortified bouillon cube program was predicted to reach the largest number of children (>29 m children over 10 years).^16^ VAS delivered via CHD would reach the second‐largest number of children (>23.6 m children over 10 years). Biofortified maize would reach the fewest children (∼13.4 m), with fortified oils reaching approximately 17.2 m children.

The number of children reached by all interventions was much larger than the number effectively covered, which in turn was much larger than the number of child deaths averted. The ranking of program impact changed as the measure of success moved from reach to effective coverage, reflecting differences in the amounts of VA delivered to children by each program. The ranking remained unchanged, however, if child deaths averted was chosen as the impact measure instead of effective coverage. The most effective VA intervention program over 10 years in terms of effective coverage (∼8.6 m CY) and lives saved (19,267) was VAS delivered via CHD. A close second was the enhanced VA‐fortified oil program, which over 10 years would effectively cover ∼8 m CY and save 15,527 lives. At the other end of the effectiveness spectrum, the biofortified maize program was estimated to effectively cover ∼2.5 m children and to save 5720 lives. The unenhanced VA‐fortified oil program and the VA‐fortified bouillon program effectively covered ∼5 and ∼7.7 m CY, respectively, and saved 9724 and 16,068 lives, respectively.

There was subnational variation for all measures of program impact, especially for reach, due primarily to variations in diets and in the consumption of fortifiable products across macroregions. Cross‐macroregional differences in effective coverage and deaths averted were also evident for all VA intervention programs, but were most notable for VAS, biofortified maize, and bouillon cubes; these differences were primarily attributable to lower VA intake in the North macroregion, and to maize consumption patterns (more common in the North macroregion).

VA intervention program costs varied substantially across the candidate programs examined in Table 1. The biofortified maize program was the least expensive (∼$1.4 m over 10 years), followed by the continuation of the low‐performing VA‐fortification program for oil (∼$2.7 m over 10 years), followed by the VA‐fortified bouillon program (∼$2.9 m over 10 years), and the improved VA‐fortified oil program (∼$4.9 m over 10 years). The most expensive program, by far, was the VAS program (∼$27 m over 10 years).

As was the case for program effects, program costs and cost‐effectiveness also varied across macroregions. For example, cost per child death averted for the VAS program varied from $3333 in the South to $643 in the North.

### Assessing the nutritional benefits and costs of combinations of VA programs

#### The BAU scenario

Cameroon already invests in two national programs to meet children's VA needs: a national VA‐fortified edible oil program^17^ and a twice‐yearly CHD program during which VAS is provided to all children between the ages of 6 and 59 months.^18^ For simulation purposes, we assumed that the current set of programs would be pursued for a decade (Y1–Y10), with the effects and costs summarized below (Table 2). On average, approximately 1.3 m children are effectively covered each year through this pair of programs. However, these programs are costly—on average, these programs cost approximately US$3 m per year.^19^

**Table 2:** Business‐as‐usual vitamin A intervention programs for preschool children in Cameroon by program, over 10 years, and annual total costs and child‐years effectively covered

	Y1	Y2	Y3	Y4	Y5	Y6	Y7	Y8	Y9	Y10
VA supplementation	SNC	SNC	SNC	SNC	SNC	SNC	SNC	SNC	SNC	SNC
Fortified edible oils (44%)	SNC	SNC	SNC	SNC	SNC	SNC	SNC	SNC	SNC	SNC
Number of child‐years effectively covered (’000s)	1198	1217	1236	1255	1274	1293	1312	1331	1350	1359
Total cost (’000s USD)	$2951	$2963	$2976	$2988	$2999	$3011	$3023	$3035	$3046	$3058

notes: SNC refers to macroregions in Cameroon: S, South; N, North; and C, Cities. All values are reported in 2013 USD.

Over a 10‐year period, the pair of VA intervention programs effectively covers approximately 12.8 m CY and costs approximately US$30 m, for an average cost of approximately US$2.34 per child‐year effectively covered (Table 3).

**Table 3:** Undiscounted 10‐year total costs and child‐years effectively covered for business‐as‐usual vitamin A intervention programs for preschool children in Cameroon, nationally and by macroregion

	National	North	South	Cities
Number of child‐years effectively covered (’000s)	12,836	6916	3503	2417
Total cost (’000s USD)	$30,051	$9968	$14,062	$6021
Cost per child‐year effectively covered (USD/child‐year)	$2.34	$1.44	$4.01	$2.49

note: All values are reported in 2013 USD.

However, these summary *national* measures of effects and cost‐effectiveness mask very substantial subnational differences (Table 3). The majority of CY effectively covered occur in the North macroregion, where VAD prevalence rates are highest and cost per effectively covered child by the VAS program is lowest. Comparing cost‐effectiveness measures across macroregions (e.g., $4.01/child‐year effectively covered in the South versus $1.44 in the North) suggests that shifting the focus of VA intervention programs toward the North macroregion may yield efficiency gains.

#### The economically optimal scenario

The results (Table 4) of the bioeconomic optimization model (aiming to cover at least the same number of CY *nationally* over the 10‐year planning time horizon more cost‐effectively than the BAU scenario, and selecting from a broader set of VA intervention programs) confirm the potential for VA intervention program efficiency gains. The enhanced fortified cooking oil program and the VA‐fortified bouillon cube program are selected by the model, even though the former does not reach peak performance until the fourth year of the simulation time horizon (Y4), and the latter faces costs but generates no benefits until Y4. Note also that the biofortified maize program is *not* selected. Although it is a relatively inexpensive program that reaches many millions of children, this intervention is predicted to effectively cover few children in this age range (given observed maize intakes and the modeled levels of maize beta‐carotene content^20^), and hence is not cost‐effective for this targeted beneficiary group. Finally, and perhaps most importantly, VAS is only selected for the North macroregion, and even there only for the first 2 years of the simulation period (again, with the optimization model objective being only to meet, at least, the BAU benefits over the same time period); the VAS program is discontinued once the benefits of the fortified oil and fortified bouillon cube programs become available to children.^21^

**Table 4:** Economically optimal set of vitamin A intervention programs for children in Cameroon over time, and their annual total costs and resulting child‐years effectively covered

	Y1	Y2	Y3	Y4	Y5	Y6	Y7	Y8	Y9	Y10
VA supplementation	N	N								
Fortified edible oils (44–72–100%)	SNC^a^	SNC^a^	SNC^a^	SNC	SNC	SNC	SNC	SNC	SNC	SNC
Fortified bouillon cubes	SNC^b^	SNC^b^	SNC^b^	SNC	SNC	SNC	SNC	SNC	SNC	SNC
Number of child‐years effectively covered (’000s)	998	1188	873	1415	1435	455	1475	1496	1516	1536
Total cost (’000s USD)	$1472	$1478	$598	$855	$855	$855	$855	$855	$855	$855

a Indicates a program year with some, but incomplete nutritional benefits.

b Indicates a program year with no nutritional benefits. SNC refers to macroregions in Cameroon: S, South; N, North; and C, Cities. All values are reported in 2013 USD.

By design, the economically optimal set of VA programs effectively covers at least as many CY as the BAU scenario (Table 5); indeed, total effective coverage is a bit higher. The economically optimal strategy retains a geographic focus on the North macroregion (51% of all effectively covered CY), but covers 757 (11%) fewer children in the North macroregion than the BAU strategy, with more CY being effectively covered in the South macroregion (180, 5%) and in the cities (152, 7%).^22^ Cost savings of this complete shift in VA intervention programs are large: total program costs over the 10‐year simulation period fall from about $30.1 m (BAU scenario) to approximately $9.5 m, and the national average cost per child‐year effectively covered falls from $2.34 (BAU scenario) to $0.71. Finally, although the number of CY effectively covered by macroregion has remained similar to that of the BAU scenario, the ratios of the macroregion‐specific average cost per effectively covered child‐year to the national average cost per effectively covered child‐year are somewhat more uniform; the model has reallocated resources across programs, across macroregions, and over time on the basis of cost‐effectiveness.

**Table 5:** Undiscounted 10‐year total costs and child‐years effectively covered for the economically optimal set of vitamin A intervention programs for preschool children in Cameroon, nationally and by macroregion

	National	North	South	Cities
Number of child‐years effectively covered (’000s)	13,086	6558	4038	2789
Total cost (’000s USD)	$9537	$4544	$3048	$1945
Cost per child‐year effectively covered (USD/child‐year)	$0.71	$0.69	$0.75	$0.70

note: All values are reported in 2013 USD.

The simulated values and their 95% intervals (in parentheses) appear in Table 6. Note in the second half of Table 6 that the three intervention programs chosen by the economically optimal solution presented in Table 4 (namely, VA‐fortified bouillon cubes, enhanced VA‐fortified edible oils, and VAS targeted to the North macroregion) appear in essentially *all* of the 1000 Monte Carlo simulation results. The competing VAS programs in the MINIMOD tool (namely, VAS in the Cities and South macroregions) are selected in only a small fraction of those simulation results, and the VA‐biofortified maize appears in slightly over 11% of these simulation results.

**Table 6:** Selected summary measures of Monte Carlo simulations of optimization model results^a^

Measure	Point estimate (95% interval) or %
Number of effectively covered child‐years over 10 years (’000s)	13,386 (12,955–14,195)
Total cost over 10 years (’000s $)	$9537 ($8709–$11,067)
Cost per child‐year effectively covered ($/child‐year)	$0.71 ($0.64–$0.82)
Monte Carlo simulation results that include the selected program (%)	
Enhanced *VA‐fortified edible oils*	100
VA‐fortified bouillon cubes	100
VAS in the North macroregion^b^	99.5
VAS in the South macroregion	0
VAS in the Cities macroregion	5.5
VA‐biofortified maize	11.3

note: All values are reported in 2013 USD.

a Optimization model simulations were run with the objective of effectively covering at least as many children as the business‐as‐usual scenario, over a 10‐year time horizon, using candidate vitamin A interventions: vitamin A–fortified edible oil or bouillon cube, high‐dose vitamin A supplements (VAS), or vitamin A–biofortified maize.

b The criterion for counting VAS as having been included in a given simulation was that the intervention appears at least three times over the 10‐year simulation time horizon.

#### Policy pathways for transitioning to more cost‐effective VA intervention programs

The MINIMOD tool identifies the *national* economically optimal strategy for implementing cost‐effective MN intervention programs. Yet, transitioning from the BAU to this lowest‐cost collection of VA intervention programs will require investments and time, and national, regional governments, and partners may have strong preferences regarding managing VAD at different spatial scales and over different time horizons. The MINIMOD tool allows policymakers to identify macroregion‐specific policy pathways and to specify alternative objectives. We present the results for three macroregions (separately), below, and also shift the focus from achieving national BAU effective coverage results to *eliminating* VAD in each macroregion.

#### The South macroregion

**Figure 1:**
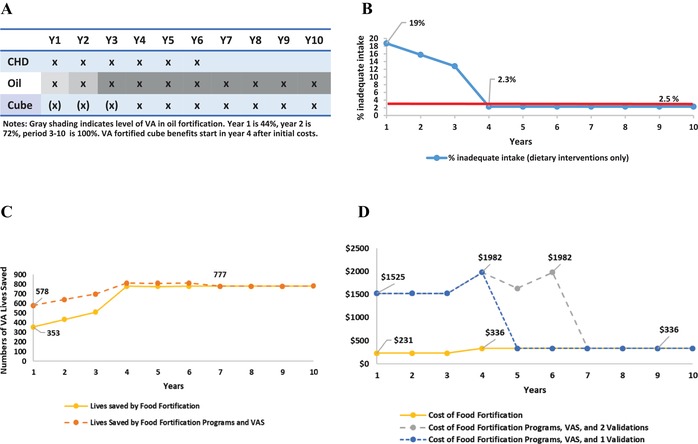
Vitamin A program intervention pathway for children 6–59 months of age in the South macroregion of Cameroon, including (A) components of the VA program over a 10‐year planning horizon, (B) prevalence of inadequate dietary vitamin A intake among children, (C) number of lives saved among children by fortification programs (yellow line) and total vitamin A programs (orange line), and (D) costs (in 2013 USD) for fortification programs (yellow line) and total vitamin A programs with a single (blue) or two (gray) validation surveys. The increasingly shaded areas associated with VA‐fortified edible oils in panel A indicate the shift from 44% to 72% to 100% (by Y3) in reaching the targeted levels of fortification. The parenthetical Xs associated with VA‐fortified bouillon cube indicate that zero nutritional benefits are generated until Y4.

In the policy pathways analysis, the VAS program is maintained in the South until Y6 (Fig. 1A), as part of the original GAVA framework involving two validation studies in nonconsecutive years (more on this, below).

At the beginning of the simulation period, 19% of children have inadequate intake (Fig. 1B). As the VA‐fortified oil program becomes more effective, this prevalence declines, but even when oil is fortified at 100% of the target amount of VA, the prevalence of inadequate intake remains above the 2.5% rate selected as the threshold to indicate adequate population VA intake. Once the benefits of VA‐fortified bouillon cubes become available (in Y4), overall VAD prevalence rates fall to 2.3%; hence, inadequate VA dietary intake is considered resolved in Y4. However, as per the GAVA protocol, VAS is continued until 2018, at which point two validation studies^23^ have been conducted (in Y4 and Y6); assuming these exercises confirm the effectiveness of the two fortification programs for reducing the prevalence of VAD, VAS is discontinued in Y7.

The total number of lives lost that are attributable to VAD in the South is approximately 780 annually (Fig. 1C), with some change over time due to increases in the under‐5 population—this represents the largest number of lives that any combination of VA intervention programs *can save*.^24^ At the outset of the simulation period, 353 lives are saved by the VA fortification programs; VAS saves approximately an additional 225 lives. As the investments in the fortification programs begin to contribute higher amounts of dietary VA, the number of lives saved by these programs increases, and reaches a plateau at approximately 777 lives saved in Y4. At that point, the VAS program's marginal contribution to lives saved is very small.

When mature, the VA food fortification programs jointly cost approximately $336,000 per year to implement, including M&E costs (Fig. 1D). VAS undertaken via CHDs costs approximately $1.3 m per year, and the validation studies cost approximately $450,000 in each of the years they are undertaken. The choice to conduct only one validation study (instead of the two surveys recommended in the original GAVA brief^25^) extends the period of programmatic savings by 2 years, eliminating 2 years of VAS and one validation study (approximately $3.0 m total).

#### The Cities macroregion

**Figure 2:**
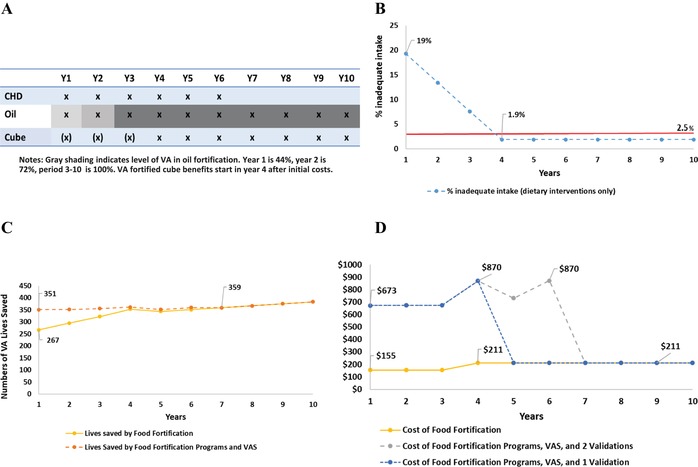
Vitamin A program intervention pathway for children 6–59 months of age in the Cities macroregion of Cameroon, including (A) components of the VA program over a 10‐year planning horizon, (B) prevalence of inadequate dietary vitamin A intake among children, (C) number of lives saved among children by fortification programs (yellow line) and total vitamin A programs (orange line), and (D) costs in 2013 USD for fortification programs (yellow line) and total vitamin A programs with a single (blue) or two (gray) validation surveys. The increasingly shaded areas associated with VA‐fortified edible oils in panel A indicate the shift from 44% to 72% to 100% (by Y3) in reaching the targeted levels of fortification. The parenthetical Xs associated with VA‐fortified bouillon cube indicate that zero nutritional benefits are generated until Y4.

The strategy plays out essentially the same way in the Cities macroregion (Fig. 2A). The VA‐fortified oil and bouillon cube programs (combined) deliver sufficient amounts of dietary VA to children to reduce the prevalence of inadequate VA intake to below 2.5% by Y4 (Fig. 2B).

The total number of children's lives lost to VAD in the Cities macroregion annually (approximately 355, Fig. 2C) is estimated to be approximately half of that of the South macroregion. In Y1, the fortification programs save 267 lives; the VAS program saves an additional 84 lives. Again, as in the South macroregion, the number of additional lives saved by VAS after the food fortification programs mature is quite small.

Because population concentrations are higher in the Cities macroregion than in the South macroregion, the costs of VAS distribution and the two validation studies are lower in the former (Fig. 2D). As was the case in the South macroregion, cost savings would accrue earlier if programmatic change is based on a single validation study as suggested by the updated GAVA framework.

#### The North macroregion

**Figure 3:**
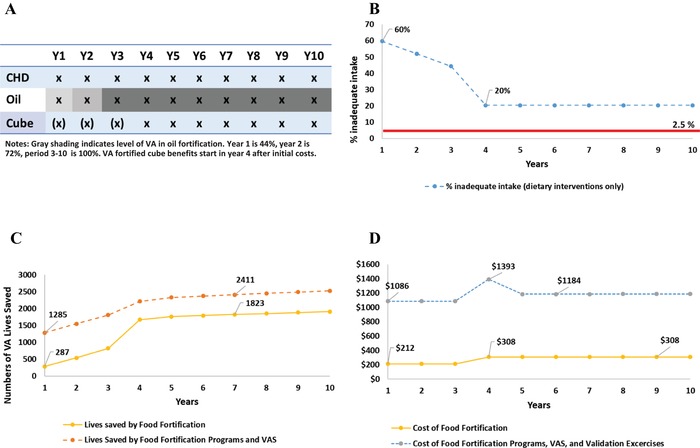
Vitamin A program intervention pathway for children 6–59 months of age in the North macroregion of Cameroon, including (A) components of the VA program over a 10‐year planning horizon, (B) prevalence of inadequate dietary vitamin A intake among children, (C) number of lives saved among children by fortification programs (yellow line) and total vitamin A programs (orange line), and (D) costs in 2013 USD for fortification programs (yellow line) and total vitamin A programs with a single (blue) or two (gray) validation surveys. The increasingly shaded areas associated with VA‐fortified edible oils in panel A indicate the shift from 44% to 72% to 100% (by Y4) in reaching the targeted levels of fortification. The parenthetical Xs associated with VA‐fortified bouillon cube indicate that zero nutritional benefits are generated until Y4.

The policy pathway is very different in the North macroregion (Fig. 3A), where the baseline prevalence of inadequate VA intake is much higher and is never completely addressed by food fortification programs included in the model. While the combined VA‐fortified oil and bouillon cube programs do considerably reduce inadequate VA intake in the under‐5 population (from 60% to 20%, Fig. 3B), they never deliver sufficient amounts of dietary VA to a sufficient proportion of the population to bring prevalence rates below the 2.5% cutoff. Therefore, the efficient 10‐year VA intervention program strategy retains VAS for children for the entire simulation period.

The estimated number of lives lost due to VAD in the North macroregion (Fig. 3C) is much larger than that in either of the other two macroregions in Cameroon. The combined food fortification programs do contribute significantly to reducing child mortality when they mature, but the VAS program continues to save hundreds of additional lives per year.

Finally, because the VAS program must be maintained throughout the 10‐year simulation period in the North macroregion, total VA intervention program costs remain high (Fig. 3D). One validation study is carried out in Y4, but it is expected to confirm model results (that prevalence of VAD among children remains unacceptably high), so a second validation study is not needed.^26^

**Figure 4:**
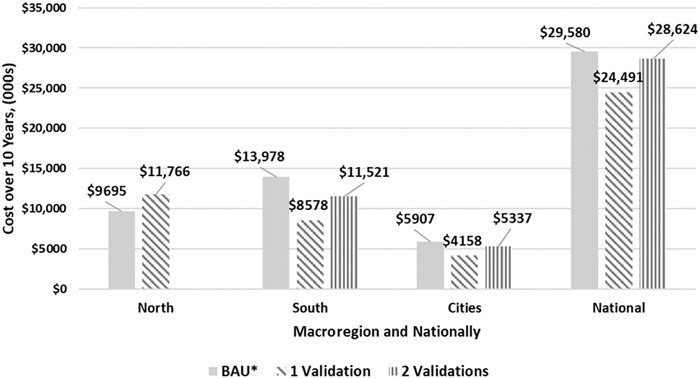
Estimated summary costs in 2013 USD of three 10‐year scenarios for vitamin A intervention programs in Cameroon, nationally and by macroregion: (A) continuing the business‐as‐usual scenario, (B) pursuing a path toward a more cost‐effective set of vitamin A intervention programs, including *one* validation survey; and (C) pursuing a path toward a more cost‐effective set of VA intervention programs, including *two* validation surveys undertaken in nonconsecutive years.

Policymakers need to know the costs associated with alternative VA intervention programs, including the macroregion‐specific costs of pursuing validation studies prior to curtailing VAS programs (if validation studies confirm that VAD is no longer a public health problem). Figure 4 provides estimates of the summary costs of three, 10‐year scenarios, nationally and by macroregion: (1) continuing the BAU scenario; (2) pursuing a path toward a more cost‐effective set of VA intervention programs, including one validation study; and (3) pursuing a path toward a more cost‐effective set of VA intervention programs, including two validation studies undertaken in nonconsecutive years, as suggested by the original GAVA framework. Nationally, maintaining the BAU set of programs is the most expensive option over 10 years. Adopting the policy pathway that includes one validation study saves approximately ∼$5 m, while the original GAVA‐recommended two‐validation pathway would save ∼$1 m over 10 years. Recall that the costs associated with the *two*‐validation‐study scenario include the costs of maintaining a VAS program for 2 years longer than the *one*‐validation‐study scenario. Note also that maintaining VAS programs while fortification programs mature, and while the results of model simulations are validated in the field, will reduce the time period during which efficiency gains associated with programmatic investments can accrue. This is especially true for the two‐validation‐study case, which allows efficiency gains to begin to accrue only in Y7 of a 10‐year modeling time horizon. Extending the modeling time horizon would increase realized efficiency gains and dramatically increase cost savings.

## Discussion

We share the global commitment to achieving sustainable, food‐based solutions to MN deficiency problems, and to identifying the policies, technologies, and institutional arrangements that will create and support them. However, in the short and medium terms, dietary intakes in the absence of large‐scale food fortification are likely to fall short of providing the amounts and types of MN required—this is especially true among children as well as women of reproductive age (WRA) in resource‐poor households in developing countries. For these populations, the fortification of staple foods can contribute significantly to achieving dietary adequacy, but in some settings, even combinations of food fortification programs will not meet needs. In such cases, direct supplementation will be required.

High‐dose VAS has been distributed through twice‐annual campaigns in LMIC for decades as a strategy to reduce child mortality. When VAS stakeholders are asked to cover the operational costs of the campaign (versus just covering the costs of providing capsules), as may become more likely as vaccination campaigns are phased out, this platform becomes quite expensive and thus unsustainable.^10^ Evidence‐based strategies are needed to allow countries to transition to medium‐ and long‐term strategies to ensure adequate VA intake and to reduce VAD‐related morbidity and mortality.

We used the MINIMOD tool and data for Cameroon to estimate the costs and likely nutritional impacts of various VA intervention programs—some of which have the potential to be implemented subnationally—on VA dietary adequacy and VA‐attributable deaths averted among young children.

Several important points emerge from the examination of the effects and costs of *individual* VA intervention programs. First, as we have shown in earlier work,^17^ the ranking of program effectiveness varies considerably depending on the measure of impact chosen, for example, selecting *reach* as a program objective will yield a very different selection of VA intervention programs than would be the case if *lives saved* were chosen as a program objective. Second, there is considerable variation in the costs associated with VA intervention programs. Third, the effects, costs, and (hence) cost‐effectiveness of *all* programs vary subnationally. These differences in cost‐effectiveness, across programs, across macroregions, and over time, suggest that efficiency gains might be achievable by shifting resources from less cost‐effective programs and macroregions to more cost‐effective programs and macroregions.

We also modeled combinations of investments in addressing VAD, for example, investing approximately $2.2 m over 3 years to improve the effectiveness of the VA oil fortification program from its baseline of 44% of targeted amount of fortification to 75% (Y2), to 100% (Y3–Y10), and investing $2.9 m in the establishment, promotion, and launching and M&E of VA‐fortified bouillon cubes, which would become available via retail outlets and (hence) begin to generate nutritional benefits in Y4. The strategy modeled also retains VAS for at‐risk children until the prevalence of inadequate VA intake falls below 2.5%, at which point inadequate VA intake would be expected to have decreased to thresholds suggesting that VAD is no longer a public health problem. The 2.5% threshold for inadequate intake was based on general targets for food fortification programs because there are no formal guidelines for the prevalence of inadequate intake that indicates a public health problem (or lack thereof), or that corresponds to a given prevalence of (biochemically determined) VAD. As estimates of dietary intake become less stable at the tails of the distribution (e.g., <10% of the population), a slightly higher threshold may also be appropriate. Specific guidance is needed on decision‐making thresholds for each type of data, as more countries seek to use dietary data to guide such programmatic decisions.

As expected, the pathways from BAU toward the more cost‐effective combinations of VA intervention programs varied by macroregion. There are some commonalities, though. First, the model selects the national enhanced VA‐fortified edible oils (over the current fortification program, in which only 44% of the target level of VA fortification is being achieved) despite its higher cost. Second, a national program to fortify bouillon cubes is chosen, despite its 3‐year start‐up period during which costs are faced but no nutritional benefits are delivered. Third, the national VA‐biofortified maize program is *not* selected—costs are relatively low, but nutritional benefits for young children are low, too.

These analyses provide concrete and practical guidance for Cameroon regarding the cost‐effective management of VAD, nationally and subnationally. They can also serve as a model for other countries to develop evidence‐based options for transitioning from short‐ to medium‐term VAD management strategies.

Importantly, these modeling results assume that intervention programs, including the M&E activities associated with them, are all operating at the levels modeled. This does not mean that we assume that programs reach 100% of the population (reach estimates are based on survey data), but we do assume that program performance is constant over time, or that it follows the performance trend included in the model. Inadequate program implementation would reduce the predicted impact on VA intake. Program implementation should be continuously monitored, and these data used to guide efforts to improve program performance. In addition, the validation studies recommended by the GAVA framework are critical to confirm that population VA status is adequate before withdrawing VAS programs. The updated GAVA framework does not specify the need for repeated validation studies before withdrawing VAS (as long as sufficient data are available to confirm adequate dietary VA intake); compared with the two‐validation framework, this approach saves field research costs and extends the timeframe for accruing programmatic savings associated with ending VAS. Finally, any programmatic changes should be accompanied by activities to monitor population status, including child mortality rates in the case of countries or regions considering withdrawing VAS programs.

### Limitations

The research underlying this paper has several limitations. First, the start‐up and ongoing costs of M&E activities associated with all VA intervention programs are included in all model simulations, and it is assumed that these M&E activities are in place and operating as intended. This is a perennial challenge in the context of developing countries, and third‐party confirmation may be required.

Second, the results presented are taken from a case study of Cameroon, an agroecologically and culturally diverse country, and consequently with a broad array of dietary intake patterns and geographic variation in MN deficiencies. Other sites may be less diverse, and their resident populations may therefore have more similar diets and prevalence rates of MN deficiencies. However, even within homogeneous agroecologies, dietary differences may exist among different socioeconomic groups, and in rural versus urban settings. Therefore, throughout other LMIC, one should expect to find dietary differences and consequently differences in the prevalence rates and severities of inadequate MN dietary intake. Regardless of the reasons for such variability, the approach adopted, and the analyses undertaken here, will generally be useful.

Third, other MN deficiencies (e.g., iron) can matter greatly for children and other beneficiary groups in Cameroon, and some of the products that could be included in this analysis (e.g., multiple MN powders) can address these deficiencies. The benefits that these products might contribute to addressing MN deficiencies other than VA are not included in this analysis—doing so would increase the chances of, but would not guarantee, their being included in the economically optimal set of MN intervention programs.

Fourth, some MN intervention programs convey other products or information that may be useful in addressing VAD. For example, in addition to VAS, CHD often provide DW tablets and guidance to mothers regarding breastfeeding, both of which can affect the VA status of children. Neither the benefits (in terms of estimated improvements in VA status) or the costs of these elements of CHD programs are included in this analysis, although previous analyses suggest that the contribution of DW tablets to improved VA status is small.^15^

Fifth, this paper focuses exclusively on young children, on VAD among them, and on the impacts, costs, and cost‐effectiveness of alternative combinations of VA intervention programs to address VAD. There are other groups in society who suffer from the consequences of VAD, for example, WRA, who would not benefit from some of the interventions addressed in this analysis (e.g., CHD) but would benefit from other programs (e.g., VA‐fortified edible oil). The cobenefits to WRA of fortification programs are not included in the analyses presented here, but if they were, they would strengthen the case for their inclusion in the cost‐effective strategy. However, VA‐biofortified maize, which the model does not select because it delivers very little dietary VA to children, may well be selected as a cost‐effective program if the focus were to shift to include WRA. These and other cobenefits are worthy of further study.

Sixth, the estimated effects of the benefits of MN intervention programs are based on detailed dietary intake data that are now somewhat dated (original data were collected in 2009), and these data do not account for seasonal variations in dietary intake. Changes in food consumption habits and breastfeeding patterns could alter baseline MN intake in ways that might affect the marginal impacts of MN intervention programs, but we do not believe that the core policy messages included in this paper would be affected.

Seventh, as with any modeling effort, it was necessary to make certain assumptions to fully populate the tool. For example, we present single estimates of lives saved (calculated using the LiST model) rather than ranges attributable to, for example, uncertainty regarding cause‐specific mortality rates at subnational level. However, model robustness tests strongly suggest that even when uncertainty regarding benefits and costs is introduced into the MINIMOD tool, the policy messages included in this paper are not affected.

Eight, we did not explore the impacts, costs, or cost‐effectiveness of alternative distribution models or platforms for VAS. This will be the focus of future work.

Finally, the costs associated with MN intervention programs included in the tool are somewhat dated (2013–2014) and these costs are held constant over the simulation timeframe, with the exception of costs that vary with the size of the target population (preschool children). In reality, these costs may vary over time, perhaps especially for CHD programs. However, sensitivity analyses suggest that core policy messages are robust to uncertainty related to costs and nutritional benefits.

### Conclusions and policy implications

Appropriate and timely data collection, analysis, and interpretation efforts are needed to document and understand the prevalence and severity of VAD, and the spatial patterns of deficiency. National and subnational perspectives and policies are needed to establish and manage cost‐effective strategies for addressing VAD.

The GAVA framework provides guidance for countries with ongoing VA fortification programs that are considering withdrawing VAS. However, for countries at an earlier stage, efforts should focus on putting in place cost‐effective strategies to address VAD. Methods and tools, such as those developed through the MINIMOD project, are needed to develop, prioritize, and strengthen national and subnational programs over time to address underlying VA intake and status.

In these simulations for Cameroon, the current edible oil fortification program is predicted to contribute to meeting VA needs of young children; improving program management can increase its effectiveness and cost‐effectiveness. Initiating a VA‐fortified bouillon cube program could also make cost‐effective contributions to addressing VAD. In the South and Cities macroregions, these programs, once mature, are predicted to reduce inadequate VA intake among children to less than 2.5%, suggesting that dietary VA intake is adequate for that population. Once these predictions are validated in accordance with the GAVA framework, VAS programs can be curtailed, since their contributions to reducing VA‐attributable child mortality will be very small. In the North macroregion, the mature food fortification programs included in this analysis are *not* predicted to reduce inadequate VA intake among children sufficiently to allow for the cessation of VAS programs.

Several important policy messages emerge. First, continuing the BAU is the costliest of the strategies explored here—all of which achieve essentially the same number of effectively covered CY and avert essentially the same number of VA‐preventable child deaths. Thus, it is worthwhile to plan the process to transition to cheaper and more sustainable strategies to address VAD.

Second, investments in and better management of VA intervention programs can significantly reduce the cost of meeting children's VA needs. For example, enhancing the performance of the VA‐fortified oil program, investing in a VA‐fortified bouillon cube program, and focusing VAS activities in the North macroregion would save over $5 m (>17% of total costs).

Third, the choice of one versus two validation studies has large fiscal consequences; for example, 2 years of additional VAS plus a second validation study in the south macroregion costs approximately US$2.9 m, but is still less expensive than continuing BAU indefinitely.

Fourth, the results presented here envision 3‐year periods for upgrading the VA‐fortified oil program, and for designing and implementing the VA‐fortified bouillon cube program. There are substantial savings associated with speeding up these processes and substantial reductions in savings associated with delays. Gains from effective M&E programs are also large. Hence, policymakers have very substantial fiscal incentives to design and implement new programs quickly, and to manage new and existing programs well.

Finally, and very importantly, detailed nationally representative data on dietary intake and biomarkers, and on MN intervention program costs, are *necessary* to identify the expected benefits and costs of alternative strategies for addressing MN deficiencies, and hence to formulate sound MN intervention policies. A recent report highlighted the lack of recent data on VAD prevalence among countries with VAS distribution programs.^29^ Without such data, the site‐specific extent of MN deficiencies is challenging to estimate, nor can cost‐effective sets of national and subnational programs be confidently designed or managed. In some settings, relatively inexpensive food fortification programs can eliminate some MN deficiencies; in other settings, more expensive combinations of programs may be required. However, without the proper underlying empirical data, policymakers cannot reasonably formulate cost‐effective strategies for solving MN deficiency problems. New approaches and tools have reduced the costs of data collection and analysis, making them more accessible to all. Collecting and analyzing such data is a policy choice and should be considered a very high priority.

## Author contributions

S.A.V., J.K., R.E.S., and K.H.B. designed the study; S.A.V., J.K., R.E.S., and K.H.B. developed methods; J.K., R.E.S., and H.L. analyzed the data and performed model simulations; and S.A.V., J.K., and R.E.S. wrote the first draft of the paper. All authors contributed to data interpretation and revisions of the paper and approved the final version.

## Competing interests

The authors declare no competing interests.

## Footnotes

1 Other programs and policies that could influence the prices or availabilities of VA‐rich foods have not been included in the MINIMOD tool to date; among other scientific gaps, convincing evidence on the impacts of these programs/policies on VA intake of young children is not yet available.

2 The present paper focuses on the original GAVA framework, but a similar approach could be used to apply the MINIMOD tool to the updated GAVA framework. However, the core results presented here and their implications for policy would not change.

3 Other candidate VA delivery vehicles could easily be introduced into the MINIMOD tool.

4 Fortification levels were taken from HarvestPlus.^26^ Assumptions regarding adoption by farmers and consumption by households are purposefully very optimistic; if this delivery platform is not cost‐effective under these assumptions, then weaker, more realistic assumptions will only reduce its cost‐effectiveness.

5 All costs are reported in 2013 USD, using an exchange rate of 500 CFA = 1 USD.

6 The incremental programmatic activities and costs associated with program improvements were identified by experts at costing workshops convened by the MINIMOD tool in Cameroon.

7 VAS program reach, as of 2009, varied by macroregion (North = 89%, South = 64%, and Cities = 58%).

8 For details of cost calculations, see Kagin *et al*.^16^

9 Funds for platforms delivering core services (e.g., polio) onto which VAS programs have been appended are disappearing. This is prompting discussions regarding the extent to which VAS and other stakeholder groups would be willing to contribute more than the marginal costs of their specific products (e.g., DW tablets) or services (e.g., breastfeeding guidance) to sustain CHD and other campaign‐based programs. In the context of Cameroon, VAS program advocates were the only groups willing to cover more than the marginal cost of product/service provision. In other words, many groups were willing to purchase a seat on the bus, but nobody was willing to pay the fuel, the driver, organizing the trip, and so on. Hence, in our calculations, the majority of overhead costs were allocated to VAS programs.

10 Model testing around this critical assumption suggested that allocating 50% or more of CHD overhead costs to VAS programs generates roughly the *same* set of cost‐effective VA intervention programs. VA‐fortified edible oils and the focus on VAS in the North macroregion remain the same, with VA‐fortified bouillon cubes and VA‐biofortified maize appearing in fewer Monte Carlo simulations than is the case when full CHD overhead costs are included in the model. As expected, the average cost per child‐year effectively covered is somewhat lower for the low‐overhead scenario ($0.68 versus $0.71). See Table 6.

11 It is not possible to know which specific children receive VAS or who consume fortified or biofortified foods/condiments and whether the same individuals would be effectively covered each year (and diets may change over time). Therefore, we use the metric “child‐years” to refer to the total number of children who are effectively covered by one of more VA intervention programs, understanding that some children might be effectively covered 1 year, and not the next.

12 The BAU strategy is, by design, a 10‐year projection of recent national VA intervention programs in Cameroon, namely: a VA‐fortified edible oils program operating at ∼44% of target levels, and the national distribution of VAS to children aged 6–59 months via CHDs.

13 The BAU and economically optimal model results presented here are slightly different from those presented in Vosti *et al*.;^17^ small upgrades have been introduced into the model, and the modest costs and nutritional benefits associated with DW tablets (distributed during CHD) have been removed.

14 This very important approach reduces economic efficiency by eliminating the potential for trading off cases of VAD that are *more* expensive to resolve in one macroregion with those that are *less costly* to resolve in another macroregion.

15 The 10‐year period is reported as Y1–Y10; all calculations and simulation model results are anchored on 2009 dietary intake data and 2013–2014 program intervention cost data. Target populations vary over time; population projections are provided by the LiST model.

16 Nearly, 94% of individuals reported consuming bouillon cubes during the 24‐h period preceding the dietary recall.

17 In the “current program” considered here, oils contained approximately 44% of target fortification levels.

18 These macroregion‐specific levels of reach are retained throughout the 10‐year model simulation period. DW tablets are also provided during these national campaigns—these tablets are not included in this analysis because the effects on VAD are modest and hence rarely appear in the list of cost‐effective VA interventions selected by the economic optimization model.

19 Increases over time in effective coverage and total costs are attributable to growth in the under‐5 population and not to changes in dietary intake or VA intervention program reach.

20 We assume ∼15 µg beta‐carotene per gram dry maize, using 12:1 equivalency. If bioconversion is higher from maize, then we would underestimate the effect of this program. That said, we do not include any adjustment for retention, and we are certainly being overly optimistic by assuming 100% replacement.

21 We did not explore alternative VAS distribution platforms; this will be the focus of future work.

22 This spatial shift in effective coverage vis‐à‐vis the BAU scenario is attributable to the increased effectiveness of the food and condiment fortification programs, which helped meet the required threshold for effectively covered children in the Cities and South macroregions, thereby reducing the number of years of VAS in the North macroregion required to meet the target for effective coverage over 10 years.

23 These are population‐based surveys undertaken to confirm that VAD is no longer occurring at a prevalence considered to be a public health problem.

24 Young children die from many causes that are not related, directly or indirectly, to VAD.^3^

25 This choice would, however, be consistent with the updated GAVA framework.

26 One could argue that no validation study is needed in the North macroregion if available data indicate that VAD is unlikely to have decreased, although conducting a nutrition survey at this point may be of use for planning further programs to address VAD. Eliminating this study would reduce costs.
